# Possible cross-infection of *Dichelobacter nodosus *between co-grazing sheep and cattle

**DOI:** 10.1186/1751-0147-54-19

**Published:** 2012-03-29

**Authors:** Torunn Rogdo, Lisbeth Hektoen, Jannice Schau Slettemeås, Hannah Joan Jørgensen, Olav Østerås, Terje Fjeldaas

**Affiliations:** 1 Norwegian School of Veterinary Science, Kyrkjevn 332/334, Sandnes 4325, Norway; 2 Animalia, Norwegian Meat and Poultry Research Centre, PO Box 396 Økern, Oslo 0513, Norway; 3 Norwegian Veterinary Institute, PO Box 750, Oslo 0106, Norway; 4 Norwegian School of Veterinary Science, PO Box 8146 Dep, Oslo 0033, Norway

## Abstract

**Background:**

The aim of this study was to investigate possible cross-infection of *Dichelobacter nodosus *in Norwegian farms practising co-grazing of sheep and cattle.

**Methods:**

Thirteen farms practising co-grazing of sheep and cattle were included in this descriptive study: five farms with a history of severe ovine footrot (Group I) and eight farms with free-stall housing of cattle and signs of mild or no footrot in sheep (Group II). Sampling for PCR detection of *D. nodosus *was performed from animals in all farms, and clinical claw examination of sheep and cattle was performed in Group II. *D. nodosus *positive samples were analysed by a multiplex PCR method that detects variants of the *fim*A gene corresponding to *D. nodosus *serogroups A through I.

**Results:**

*D. nodosus *serogroup A was identified more frequently in sheep from farms with a history of severe footrot (Group I) versus from Group II, and in most of the farms with a history of severe footrot there was a coexistence of *D. nodosus *serogroup A in sheep and cattle. In one farm heel horn erosion and dermatitis emerged in cattle after co-grazing with sheep suffering from severe footrot where *D. nodosus *serogroup A was detected. Six months later heel horn erosion and dermatitis were still diagnosed, and *D. nodosus *serogroup A was identified. Out of the 16 *D. nodosus *positive sheep samples from Group II, ten of the samples were positive by the *fim*A serogrouping PCR. Among these 10 samples all serogroups except G were detected. All the *D. nodosus *serogroups detected in sheep were also present in the corresponding cattle herds.

**Conclusion:**

The clinical findings and the coexistence of the same serogroups in co-grazing sheep and cattle could indicate cross-infection. However, further research including isolation of the bacterial strains, virulence-testing and genetic identification, is needed.

## Background

Ovine footrot is a major cause of lameness in sheep worldwide [1]. For 60 years the disease was considered eradicated in Norway, but in the spring of 2008 footrot was reported [2]. Until then, ovine foot problems were paid relatively little attention to by farmers and veterinarians, although lameness had been a main reason for culling in some flocks [3]. In a flock health study performed in 2007-2008, *Dichelobacter nodosus *was detected by PCR in two sheep flocks with mild or no clinical symptoms and in one flock where several animals were suffering from lameness [3]. This initiated surveillance and clinical examination of 3300 sheep flocks where animals from about 1000 flocks were sampled [4,5]. *D. nodosus *was detected by PCR in more than 500 of these flocks, whereas only about 50 flocks were severely affected. The majority of the *D. nodosus *positive flocks had mild or no symptoms of footrot.

Ovine footrot begins as an interdigital dermatitis that may progress to necrotic separation of the claw capsule from underlying tissues. *D. nodosus *is the main causative agent and may act in synergy with *Fusobacterium necrophorum*, although recent studies by Witcomb et al. indicate that the primary influence of *F. necrophorum *may have been overestimated [6]. Based on antigenic variation of the type IV fimbriae, ten serogroups and 18 serotypes of *D. nodosus *have been described [7,8]. The virulence of the different strains is, however, not linked to serogroup identification, but to the bacterial strains' ability to produce extracellular proteases [9].

*D. nodosus *and *F. necrophorum *are also important etiological agents for the bovine contagious claw diseases interdigital dermatitis and interdigital phlegmon. In addition *D. nodosus *is considered to play a synergetic role in the development of bovine digital dermatitis primarily caused by *Treponema *spp. [10]. The incidence of the bovine contagious claw diseases including interdigital dermatitis/heel horn erosion, digital dermatitis and interdigital phlegmon, have increased in Norway over the last years [11], [Terje Fjeldaas, personal communication]. In a survey performed in 2002 the prevalences of dermatitis and heel-horn erosion in free-stall herds were 7% and 40%, respectively [12]. Interdigital dermatitis has been prevalent in Norwegian free stall cattle herds for some years [13], but infection with *D. nodosus *in cattle was not diagnosed until recently. Following the increased interest in ovine footrot and the new availability of diagnostic tools, *D. nodosus *has been detected in several Norwegian cattle herds.

Co-grazing is common in Rogaland County in Norway where the density of both sheep and cattle is the highest [14] and where the climate is among the most humid in Norway [15]. There is limited knowledge as to how this may contribute to the distribution of severe ovine footrot (SFR) and as to whether cattle can transfer virulent *D. nodosus *to sheep under these conditions. Few studies have investigated transfer of different *D. nodosus *strains from sheep to cattle and vice versa.

The aim of this study was to assess contagious claw diseases in cattle and footrot in sheep in farms where these two species share the same pastures, to identify *D. nodosus *serogroups and to investigate whether cross-infection of *D. nodosus *could have occurred.

## Methods

### Study design and selection procedure

Farms practising co-grazing of sheep and cattle where *D. nodosus *was expected to be widespread, were selected. *D. nodosus *is widespread in sheep flocks with SFR, and farms with SFR were selected for Group I. *D. nodosus *is also expected to be widespread in free stall housed dairy cattle herds where interdigital dermatitis and heel horn erosion are prevalent [11], and farms with free-stall housing of cattle and corresponding sheep flocks showing mild or no symptoms of footrot were selected for Group II.

### Study population

Thirteen mixed farms in Rogaland County in the south west of Norway were included in the study. The farms keep meat sheep and dairy cattle, 1451 sheep and 663 cows in all. All farms practised co-grazing of the animals.

Five farms practising co-grazing counting a total of 900 sheep and 244 cows and where SFR had been diagnosed during the summer of 2008, were included in Group I (Farm 1-5). In Nov/Dec 2008, when most of the recording and sampling were done, no farms where cattle co-grazed with sheep suffering from SFR were available, since measures to control and reduce the disease in sheep had already been initiated through the elimination programme "Healthy Feet" [4]. Results from previous sampling of sheep in these farms were therefore used. The number of sheep samples and the time of sampling varied between the farms. The farm data for this group are listed in Table 1.

**Table 1:** Data from 13 Norwegian farms where sheep and cattle were co-grazing in 2008

Farm	Sheep		Cows		Housing	Co-grazing	
			
	Flocksize, winter^1^	Breed	Herd size^1^	Breed		Only heifers/dry cows	All cattle
Group I							
1^a^	180 (4)	NKS	16 (10)	NRF	Tie stall		x
2^a^	280 (5)	NKS	89 (18)	NRF	Free stall	x	
3^a^	270 (7)	NKS	85 (10)	J	Free stall	x	
4^a^	150 (10)	NKS	41 (10)	NRF	Tie stall	x	
5^a^	20^2 ^(17)	NKS	13 (10)	NRF	Tie stall		x
Group II							
6^b^	35 (10)	NKS	77 (10)	NRF/H	Free stall	x	
7^b^	25 (10)	NKS	38 (10)	NRF	Free stall		x
8^b^	50 (10)	NKS	27 (10)	NRF	Free stall		x
9^b^	46 (10)	NKS	33 (10)	NRF/H/J	Free stall	x	
10^c^	50 (10)	NKS	45 (10)	NRF	Free stall		x
11^c^	45 (10)	NKS	65 (10)	NRF/H	Free stall	x	
12^c^	100 (10)	NKS	68 (10)	NRF/H	Free stall	x	
13^c^	200 (10)	NKS	67 (10)	NRF/H	Free stall	x	

^1^Number of samples in brackets

^2^20 sheep bought from farm 3 and 4 winter 2008

^a^Severe footrot previously recorded in sheep flock

^b^Mild footrot previously recorded in sheep flock

^c^No footrot previously recorded in sheep flock

NKS = Norwegian White Sheep, mainly

NRF = Norwegian Red Cattle

H = Holstein

J = Jersey

Eight other farms practising co-grazing, free-stall housing of cattle all in all counting 551 sheep and 419 cows and having sheep showing mild or no symptoms of ovine footrot, were included in Group II (Farm 6-13). Other herd data for this population are listed in Table 1. In four of the farms symptoms of mild ovine footrot (MFR) had recently been recorded, and the presence of *D. nodosus *in the interdigital space of sheep had been confirmed by PCR [4]. No symptoms of MFR had been found in the sheep of the other four farms. The cattle - sheep contact varied between the farms. In some of the farms the sheep co-grazed with all of the cattle, in others only with heifers and dry cows. At the time of sampling, Nov/Dec 2008, the cattle had been kept in free stall barns for at least two months, and consequently there had been time for the bacteria to spread between the cows. The cattle had no contact with sheep during the housing period.

### Recording of data and sampling for PCR

**Figure 1:**
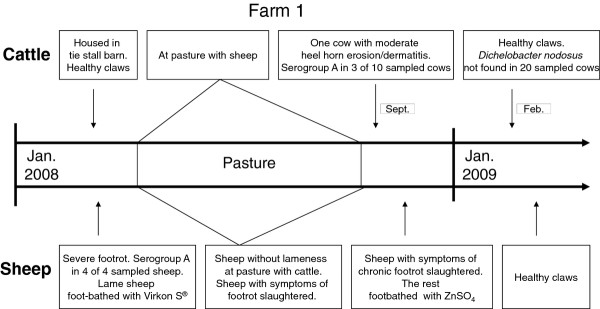
**Events in farm 1 indicating cross-infection of *Dichelobacter nodosus *from sheep with severe footrot to cattle co-grazing at pasture**.

**Figure 2:**
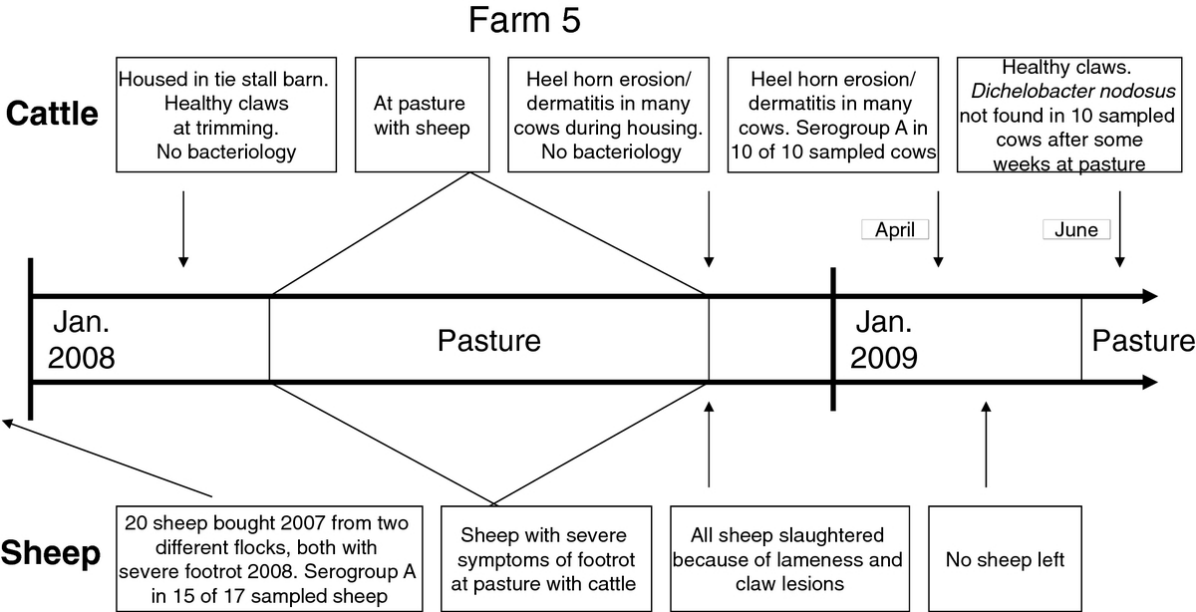
**Events in farm 5 indicating cross-infection of *Dichelobacter nodosus *from sheep with severe footrot to cattle co-grazing at pasture**.

In Group I samples from 43 sheep were taken during the summer of 2008. The samples from 58 cattle were taken during the winter/spring of 2008/2009. The cows in farms 1 and 5 were sampled an additional time in order to follow the events in these farms as depicted in Figure 1 and 2. A total of 88 samples were taken from cattle in Group I. A sterile wooden stick was used to scrape the interdigital area, and the stick was subsequently placed in Peptone Buffered Saline (PBS) containing EDTA. The sheep samples were collected from the feet with clinical symptoms, whereas the cow samples were always collected from the right hind foot. They were sent to the laboratory by mail. The samples were taken without systematic clinical recording of claw disorders, but information on chronological events regarding claw health in these farms between January 2008 and April 2009 was gathered from the Norwegian Sheep Health Service and from telephone conversations with the farmers.

In Group II clinical examination and sampling for PCR analyses for *D. nodosus *of ten cows and ten ewes in each of the eight farms were performed at claw trimming of the cattle. Animals with lameness or claw disorders previously noted by the farmer were selected, otherwise randomly. The examination included recording of the contagious claw lesions heel horn erosion (HHE) and dermatitis (D) in cattle and symptoms of MFR and SFR and also abnormal claw shape (ACS) and white line fissure (WLF) in sheep. The definitions for the recorded claw disorders in cattle and sheep are adapted from Sogstad [12] and Stewart & Claxton [16], respectively, and they are listed in Table 2.

**Table 2:** Definition of recorded claw disorders/diseases in sheep and cattle

Lesion	Abbreviation	Score	Definition
Mild footrot^s^	MFR^1^	1,2	Hairless, hyperemic, slightly exudative lesion of the interdigital space without under-running of horn
Severe footrot^s^	SFR^2^	3-5	Gradually increasing under-running and separation of sole starting from the interdigital space and gradually spreading to the wall
Abnorm claw shape^s^	ACS	**-**	All claw shapes except normal and overgrown claws with otherwise normal shape
White line fissure^s^	WLF	**-**	Separation in the white line between the sole and the wall
Heel horn erosion^c^	HHE	-	V-shaped fissures and craters of the heel/bulb
Dermatitis^c^	D	1	Superficial, hyperemic, slightly exudative lesion of the digital/interdigital skin
		2	Exudative, slightly ulcerative lesion with thickening of the skin
		3	Ulcerative, spontaneously bleeding lesion with thickening of the skin and great pain

^s ^= sheep

^c ^= cattle

^1^MFR includes lesions of score 1 and 2, according to the Australian system for scoring of footrot [16]

^2^SFR includes lesions of score 3, 4 and 5 [16]

### PCR analyses

Laboratory analyses were performed at the Norwegian Veterinary Institute in Oslo. Template DNA was extracted from the swab-sticks in PBS with EDTA, using a NucliSens^®^easyMAG™ extraction robot (bioMérieux, Marcy l'Etoile, France) according to the manufacturer's instructions. Extracted DNA was stored at - 80 C. First a PCR method to detect *D. nodosus *was performed [17]. In this method a *D. nodosus *specific fragment of the 16S rRNA gene is amplified. Positive samples were further analysed by a multiplex PCR which detects variants of the *fim*A gene corresponding to serogroups A through I [7]. All PCR products were visualised and captured under UV light following gel electrophoresis. Negative and positive controls were used for all PCR reactions. Initially DNA extracted from control strains kindly provided by the University of Bristol was used as positive controls. Simultaneously, culturing of *D. nodosus *was started, and further sequencing of the *fimA *gene in a number of isolates in order to use these as controls (serogroups sequenced A, B, C, E and I), was performed. These in-house strains were subsequently used for control.

## Results

### Farms with a history of severe ovine footrot (Group I)

Results from PCR analyses are presented in Table 3. *D. nodosus *serogroup A was detected frequently in farms with a history of SFR and was present in samples from all five sheep flocks, whereas serogroup H was present in four of them. Serogroup A was also found in four of the five corresponding cattle herds and serogroup H in one of them. In farm 1 *D. nodosus *serogroup A was detected in claw samples from cattle after co-grazing with healthy sheep from a flock previously treated for SFR and where *D. nodosus *serogroup A had been detected (Figure 1). In farm 5 HHE and D were diagnosed in cattle for the first time after co-grazing with sheep suffering from SFR where *D. nodosus *serogroup A was detected (Figure 2). At claw trimming six months later, at the end of the housing period (April), HHE and D were still diagnosed in many cows, and serogroup A was found in all samples taken from ten cows. However, new samples taken at pasture in June were negative, and at claw trimming performed by a professional trimmer at the end of the grazing season the cows were reported to have healthy claws.

**Table 3:** Animals (n) with different serogroups (A to I) of *Dichelobacter nodosus *identified by PCR analyses in the sheep flocks and the corresponding co-grazing cattle herds

Farm	Species	Frequency of serogroup-PCR- positive samples	Animals (n)								
Group I			A	B	C	D	E	F	G	H	I
1^a^	C	3/10	**3**^1^	0	0	0	0	0	0	0	0
	S	4/4	**4**	0	0	0	0	0	0	0	0
2^a^	C	14/18	**2**	12	10	0	8	0	0	0	0
	S	5/5	**5**	0	0	0	0	0	0	2	0
3^a^	C	6/10	0	0	0	0	0	10	0	**6**	0
	S	7/7	5	1	2	0	0	0	0	**3**	2
4^a^	C	3/10	**3**	0	0	0	0	0	0	0	0
	S	10/10	**10**	0	0	2	2	2	0	7	0
5^a^	C	10/10	**10**	0	0	0	0	0	0	0	0
	S^2^	17/17	**15**	1	2	2	2	2	0	10	2
Group II											
6^b^	C	8/10	6	2	3	0	0	0	0	0	**4**
	S	1/10	0	0	0	0	0	0	0	0	**1**
7^b^	C	10/10	0	**10**	7	1	1	1	0	5	**8**
	S	2/10	0	**2**	0	0	0	0	0	0	**1**
8^b^	C	10/10	0	1	1	**1**	**9**	**1**	0	**5**	2
	S	1/10	0	0	0	**1**	**1**	**1**	0	**1**	0
9^b^	C	10/10	**6**	8	7	0	0	0	0	0	2
	S	1/10	**1**	0	0	0	0	0	0	0	0
10^c^	C	10/10	1	8	2	1	8	1	0	9	1
	S	0/10	0	0	0	0	0	0	0	0	0
11^c^	C	10/10	0	**3**	**2**	6	2	4	0	6	**3**
	S	3/10	0	**2**	**1**	0	0	0	0	0	**1**
12^c^	C	10/10	6	0	3	0	1	0	7	9	3
	S	0/10	0	0	0	0	0	0	0	0	0
13^c^	C	10/10	0	4	**6**	3	0	0	2	**9**	4
	S	2/10	0	0	**1**	0	0	0	0	**1**	0

C = cattle

S = sheep

^a ^Severe footrot previously recorded in sheep flock

^b ^Mild footrot previously recorded in sheep flock

^c ^No footrot previously recorded in sheep flock

^1 ^Positive sheep samples and the corresponding

positive cattle samples in the co-grazing farms are written in bold

^2 ^20 sheep bought from herd 3 and 4. SFR diagnosed after delivery of sheep

### Farms with free-stall housing of cattle and no or mild ovine footrot (Group II)

Clinical recordings are presented in Tables 4 and 5. Sixteen out of 80 samples from sheep were *D. nodosus *positive by PCR, and ten of these samples produced a result in the serogrouping PCR (Table 3). All serogroups except G were detected. Five of the *D. nodosus *positive sheep samples came from farms 11 and 13 where no symptoms of footrot had been recorded. All the serogroups present in sheep were also present in the corresponding cattle herds. In the co-grazed cattle herds 78 of 80 samples were *D. nodosus *positive by PCR. Serogroups identified in the eight herds are presented in Table 3. Samples with only one serogroup were identified from 12 cows, whereas 2 and ≥3 serogroups were identified in samples from 24 and 39 cows, respectively. All nine serogroups, A-I, were present, but differed between the farms. Serogroup A was detected in six cows in three different farms and in one cow in one farm, but in the rest of the herds serogroup A was absent. The serogroup found most frequently, serogroup H, was not found at all in two of the farms.

**Table 4:** Recorded claw disorders in 80 co-grazed sheep in farms with no or mild footrot^1^

Farm	n	MFR	SFR	ACS	WLF	NCD
6^a^	10	3 (1)	0	0	3 (1)	4 (2)
7^a^	10	0	0	0	1 (1)	9 (2)
8^a^	10	2	0	0	0	8 (1)
9^a^	10	0	0	3	3 (2)	6 (1)
10^b^	10	0	0	3	0	7
11^b^	10	2 (1)	0	0	2	7 (2)
12^b^	10	0	0	1	0	9
13^b^	10	0	0	0	0	10 (2)

MFR = Mild footrot

SFR = Severe footrot

ACS = Abnorm claw shape

WLF = White line fissure

NCD = Number of sheep with no claw disorders

^1^Serogroup-PCR-positive samples (16) in brackets

^a^Mild footrot previously recorded in sheep flock

^b^No footrot previously recorded in sheep flock

**Table 5:** Recorded contagious claw diseases in 80 co-grazed cows in farms with no or mild ovine footrot^1^

Farm	n	HHE	D 1	D 2	D 3	NCCD
6^a^	10	0	1	2	0	7
7^a^	10	0	2	1	0	7
8^a^	10	2	1	2	0	7
9^a^	10	1	2	0	0	8
10^b^	10	8	4	6	0	0
11^b^	10	6	5	1	0	4
12^b^	10	6	1	2	0	4
13^b^	10	6	3	3	0	4

HHE = Heel horn erosion

D = Dermatitis, graded from mild to severe (1-3)

NCCD = Number of cows with no contagious claw diseases

^1^Serogroup-PCR-positive samples in 78 of 80 cows

^a^Mild footrot previously recorded in sheep flock

^b^No footrot previously recorded in sheep flock

## Discussion

### General considerations

The fact that all clinical findings were recorded by the first author, ensured comparable data. It was, however, a disadvantage for our study that the sheep flocks in Group I were already treated with antibiotics, foot bathed or discarded as advised by the "Healthy feet" programme [4] at the time of recording. Systematic individual recording of clinical findings in these flocks/herds was not possible.

The *fim*A PCR was performed directly on DNA from swab samples. This PCR method has mainly been tested on template DNA from pure *D. nodosus *isolates and only on a small number of swab samples from the interdigital cleft of sheep [7]. Since serogrouping/-typing can not be used to predict virulence in *D. nodosus *[8], it would have been an advantage to cultivate and virulence test *D. nodosus *isolates. But these methods were not established in Norway at the time of the study, and data regarding clinical manifestations in sheep were used as an indication of bacterial virulence. Preliminary results from a cultivation study show that several different *D. nodosus *serotypes are rarely found in sheep flocks and cattle herds in Norway. We can not exclude non-specific reactions in the PCR-method (Hannah Joan Jørgensen, personal communication).

Analyses for *F. nechrophorum *and *Treponema *spp. could have been informative, but were not considered necessary, since the aim of this study was to investigate whether *D. nodosus *could be spread between sheep and cattle. Consequently and also for practical and financial reasons, we decided only to analyse for *D. nodosus*.

### Possible spread of ovine footrot-causing *D. nodosus *to cattle (Group I)

Virulence is not linked to serogroup identification, but the finding in this study, that *D. nodosus *serogroup A seems to be associated with severe ovine footrot, is in agreement with a recent study indicating that the majority of virulent *D. nodosus *isolated from sheep in Norway belongs to serogroup A (manuscript in preparation). But benign *D. nodosus *belonging to serogroup A has also been detected.

Heel horn erosion and D seemed to emerge in cattle for the first time in farm 5 after grazing with sheep diagnosed with SFR and *D. nodosus *serogroup A. This is unexpected since these bovine claw diseases are usually reduced or disappear during grazing at pasture [12,18]. There was a coexistence of *D. nodosus *serogroup A in sheep and cattle in these herds, and *D. nodosus *serogroup A was still found in samples after several months, although the cows in this farm were housed in a tie stall barn where these diseases usually occur with low prevalence. These findings may indicate that *D. nodosus *serogroup A has indeed been transferred from sheep with SFR to cattle. However, since a serogroup may include several serotypes, there may have been different strains of *D. nodosus *causing infections in different animals.

Animal trade and shared pastures are potential risk factors for spreading of ovine footrot [19]. Internationally footrot in sheep is controlled at flock level, and precautions are taken to avoid new infections from other flocks. According to Greenough bovine interdigital dermatitis and ovine footrot are, however, caused by different variants of *D. nodosus*, and transmission from sheep to cattle of bacterial strains causing SFR has not been reported [10]. *D. nodosus *has been isolated from clinically normal cattle [20], but cattle is not considered a source of virulent *D. nodosus *in sheep [10]. Indirect transmission of *D. nodosus *between sheep on unimproved pasture, which has resulted in virulent ovine footrot, has been reported [21]. Early studies by Egerton & Parsonson indicated that experimental transmission of *D. nodosus *from cattle to sheep can result in ovine footrot [22]. Natural transmission was not demonstrated in their test, but it was suggested that cattle is a potential source of infection in sheep. Natural transmission of *D. nodosus *between a steer with dermatitis and sheep was attempted by Wilkinson et al., but the symptoms in sheep were only of benign character [23]. Also later trials did not succeed in experimental transmission of virulent strains of *D. nodosus *from sheep to cattle [20].

Spread of ovine footrot can occur by introduction of infected animals to a flock or by animals from one flock contaminating the environment of another [24]. *D. nodosus *has been shown to survive on pasture for 7 days [19], but transmission through pastures contaminated more than 1 day earlier is unlikely [9,18]. Environmental and management factors influence the outcome of infection. A mean daily temperature of 10°C and high humidity promote the development of the disease [25]. Such conditions were more or less present in the region where the study population was located, and our study indicates that variants with the ability to cause SFR also may have been transferred between cattle and sheep on pasture.

### Possible spread of bovine interdigital dermatitis-causing *D. nodosus *to sheep (Group II)

Although there were rather few cases and the clinical lesions were mild, there seemed to be an association between farms with *D. nodosus *positive sheep and clinical findings in our study. When comparing all *D. nodosus *positive and -negative flocks (six and two flocks, respectively), there was an association between clinical findings and the detection of *D. nodosus*. Five of the six positive flocks had a total of 19 claw disorders, MFR, ACS and WLF, whereas only four sheep with ACS were recorded in the two *D. nodosus-*negative flocks (Table 4). These findings could be explained by a connection between bacteriology/clinical findings and environmental causal connections or a combination of these two.

As expected, HHE and D were prevalent in the free stall cattle herds. Poor hygiene with manure covering the claw horn is detrimental to claw horn and is a major problem in many free stall herds [26,27]. This is the most important predisposing factor for contagious bovine claw diseases [28], and *D. nodosus *was present in almost all samples from the free stall cattle herds. There was probably a risk for cross-infection to sheep when these cattle were let straight out on pasture with feet ingrained with manure from barns potentially contaminated with *D. nodosus*. In some of the farms in Group II only heifers and dry cows co-grazed with sheep.

In Group II all the *D. nodosus *serogroups found in the sheep flocks were also found in the co-grazed cattle herds, whereas several of the serogroups found in cattle were not found in the corresponding sheep flocks. This may be a consequence of greater genetic diversity among *D. nodosus *isolates from cattle than from sheep. It could also indicate that bovine *D. nodosus *had been transferred to sheep. Still we can not disregard the possibility that detection of the same *D. nodosus *serogroups in co-grazed sheep and cattle could be coincidental.

## Conclusion

In our study of Norwegian farms practising co-grazing of sheep and cattle, *D. nodosus *belonging to serogroup A was prevalent in sheep in farms with a history of severe footrot (Group I). The study also demonstrates the coexistence of *D. nodosus *serogroup A in sheep and cattle, and the study of events indicates that the infection lasted longer than 6 months in one cattle herd. In the study of farms practicing free-stall housing of cattle and having sheep showing symptoms of mild or no ovine footrot (Group II), all the serogroups found in the sheep flocks were also found in the corresponding cattle herds. The clinical findings and the coexistence of the same serogroups in co-grazing sheep and cattle indicate cross-infection. However, further research including isolation of the bacterial strains, virulence-testing and genetic identification, is needed.

## Competing interests

The authors declare that they have no competing interests.

## Authors' contributions

TR contributed to the design of the study. She visited the farms and performed all the clinical recordings and sampling for PCR analyses. She performed the data analyses and also made the draft of the manuscript. LH contributed to the study design, the selection of farms, the analyses of data and the writing of the manuscript. JS established the laboratory methods and performed the analyses. HJJ contributed to data interpretation and to writing of the manuscript. TF contributed to the design of the study and to the writing of the manuscript. He was the main supervisor for TR in the present study during the field recordings and sampling, the analyses of the data and the writing period. All authors read the manuscript several times and approved the final manuscript.
